# Status and associated factors of gerontological nurse specialists’ core competency: a national cross-sectional study

**DOI:** 10.1186/s12877-023-04153-0

**Published:** 2023-07-21

**Authors:** Hongxiu Chen, Lihui Pu, Shengyuan He, Xiuying Hu, Qian Chen, Zhaojing Huang, Linan Cheng

**Affiliations:** 1 West China School of Nursing, Innovation Center of Nursing Research, Nursing Key Laboratory of Sichuan Province, West China Hospital, Sichuan University, Chengdu, China; 2 Menzies Health Institute & School of Nursing and Midwifery, Griffith University, Brisbane, Australia; 3 Innovation Center of Nursing Research, Nursing Key Laboratory of Sichuan Province, West China Hospital, Sichuan University, Chengdu, China; 4 Innovation Center of Nursing Research, Nursing Key Laboratory of Sichuan Province, Center of Gerontology and Geriatrics, West China Hospital, Sichuan University, Chengdu, China

**Keywords:** Geriatric nursing, Specialist nurses, Core competency, Cross-sectional study, Influencing factors

## Abstract

**Background:**

Nurses’ core competency directly affects patients’ safety and health outcomes. Gerontological nurse specialists play an essential role in improving older adults’ health status. However, little is known about their core competency level and the factors influencing core competency. Therefore, this study aimed to investigate the status of core competency and factors influencing the core competency of gerontological nurse specialists in China.

**Methods:**

A multicenter cross-sectional study was conducted on gerontological nurse specialists certified by province-level or above organizations across China between March 2019 and January 2020. The Revised Core Competency Evaluation Instrument for Gerontological Nurse Specialists was used to measure participants’ core competency. The median, frequencies, and percentages were used to describe participants’ characteristics and level of core competency. Multivariate stepwise regression analysis was applied to analyze the factors influencing core competency.

**Results:**

The median score of gerontological nurse specialists’ core competency was 3.84, and professional development skills and research and analysis decision-making skills had the lowest scores among the dimensions. The multivariate stepwise regression analysis showed that individual-level factors (i.e., working experience length of geriatric nursing and attitudes toward caring for older adults), employer-level factors (i.e., departments, job responsibilities, the degree of satisfaction toward the attention and support and the promotion rules provided by the hospital or department), and training-associated factors (i.e., economic zone where training organizations are located and the degree to which the training content met clinical needs) are independently associated with gerontological nurse specialists’ core competency level (P < 0.05).

**Conclusions:**

This study showed that gerontological nurse specialists’ core competency needs further improvements, especially regarding professional development skills and research and analysis decision-making skills. Additionally, individual-, training-, and employer-level factors could influence their core competency level, indicating that interventions targeting these factors could be applied to improve the core competency of gerontological nurse specialists.

## Background

The elderly population is increasing worldwide and is set to double from 1.05 billion people aged over 60 years in 2020 to 2.08 billion in 2050 [1]. Moreover, during this period, the aging population in developing countries will increase by 250%, compared to 71% in developed countries. This indicates that the aging situation in developing countries is more severe [2]. Older adults, who account for the majority of all hospitalizations and utilization of health services, often have multimorbidity, polypharmacy, and decreased personal autonomy, making their health problems more complex and complicating their care [2]. Therefore, gerontological nurse specialists have emerged to address the complicated health problems arising by older adults and have already been trained in many countries and regions globally, such as the United States, Australia, New Zealand, Japan, Hong Kong, and mainland China.

However, the definition, admission requirements, and training programs of gerontological nurse specialists are quite different among countries. In the Chinese context, gerontological nurse specialists are defined as registered nurses who have passed a qualification examination and obtained certification after finishing a training program for gerontological nurse specialists [3]. The most common admission requirements for registered nurses to attend the training program are as follows: (1) having at least a college degree in nursing, (2) having at least a senior nurse professional title, and (3) having at least two years of working experience in geriatric nursing. The training programs for gerontological nurse specialists, provided by nursing associations at or above the provincial level, are short-term continuing education that lasts for two to three months (i.e., one month for geriatric nursing-related theory curriculum and one to two months for specialized geriatric clinical practice). Compared with developed countries such as the United States, which train gerontological nurse specialists in the format of a degree education and require them to have a master’s degree or clinical doctor’s degree, the training of gerontological nurse specialists in China is lagging behind comparatively; however, this is in line with the current situation of nursing education in China because postgraduate nursing education has not yet been popularized in China, and geriatric nursing has only been valued and developed in the last ten years.

Nurse competency is important because it directly affects patients’ safety and health outcomes as well as the quality and effectiveness of nursing services [4, 5]; therefore, many studies are actively exploring the competency of nurses in different nursing specialties, in various jobs, and at various levels [6–15]. Studies on gerontological nurse specialists’ competency have also been conducted, and they mainly focus on exploring the core competency requirements and developing core competency frameworks or core competency assessment tools [3, 16–19]; meanwhile, a paucity of studies have explored the status of and factors influencing gerontological nurse specialists’ core competency [20]. It is essential to evaluate nurses’ level of competency and grasp the factors that potentially affect competency to allow nurses to self-reflect and provide valuable information for managers and educators to design continuing education programs and other measurements aiming to promote nurse competency [21, 22].

Many methods can measure nurses’ competency, such as standardized examinations, standardized patients, self-evaluation, and peer review. Self-evaluation is one of the most frequently used and available methods [23–27]. Since 2013, mainland China has trained more than 2,500 gerontological nurse specialists; however, little is known about their core competency level and the factors that might affect core competency. Therefore, this study aimed to investigate the core competency level of gerontological nurse specialists across China through self-evaluation and explore the related factors affecting their core competency from the perspectives of individual-, employer-, and training-related factors. The findings of this study might provide more insight into improving the training system of gerontological nurse specialists and provide direction for improving their core competency, especially for countries with similar training systems for gerontological nurse specialists.

## Methods

### Study design and setting

A multicenter cross-sectional study was conducted on gerontological nurse specialists certified by province-level or above organizations across China.

### Participants

Gerontological nurse specialists who met the following criteria were included: (1) trained by province-level or above training organizations; (2) certified as the gerontological nurse specialist after January 2014; and (3) participating in this survey voluntarily. Gerontological nurse specialists who met the following criteria were excluded: (1) cannot complete the online questionnaire for various reasons, or (2) no longer working in any medical institution during the survey period.

### Measurements

#### Potential variables related to the core competency of gerontological nurse specialists

Systematic literature reviews of studies exploring the factors associated with the core competency of all kinds of geriatric nurses and nurse specialists in other fields were performed to identify factors that may affect gerontological nurse specialists’ core competency. Based on the results of the literature review and consultation with three experts with more than 20 years of experience working in geriatric nursing, we selected the potential associated factors and categorized them into three levels: individual-, employer-, and training-level factors. The details of the potential associated factors collected in this study are shown in Table 1.

**Table 1:** Variables included in the questionnaire

Variables and measure methods
∎ **Individual-related factors**
- Age, gender, marital status, education level, job title, professional title, working experience lengths of geriatric nursing - Attitudes toward caring for older adults (evaluated by a single-item Likert-type scale, with − 5 indicating that participants feel negatively toward caring for older adults, 0 indicating that participants neither feel negatively nor passionately toward caring for older adults, and 5 indicating that participants feel passionately toward caring for older adults)
∎ **Employer-related factors**
- Hospital level, department, whether the department had exact job responsibilities for gerontological nurse specialists, and whether the gerontological nurse specialist certificate was associated with a priority for promotion or increase in salary - The degree of satisfaction toward the attention and support, learning opportunities, promotion rules, and position salary or allowance provided by the hospital or department (evaluated by a single-item Likert-type scale, with 1 indicating completely unsatisfied and 5 indicating completely satisfied)
∎ **Training-related factors** - Location of the gerontological nurse specialist training organizations, the degree to which the training content met clinical needs (evaluated by a single-item Likert-type scale, with 1 indicating completely unsatisfied and 5 indicating completely satisfied), and whether they participated in continuing education training related to geriatric nursing after obtaining the gerontological nurse specialist certificate.

#### Instrument for evaluating the core competency of gerontological nurse specialists

The revised version of the Core Competency Evaluation Instrument for Gerontological Nurse Specialists was used to evaluate core competency. This original instrument was initially developed based on multi-methodologies, including a systematic literature review, theoretical analysis, and the Delphi method (28 experts from 21 tertiary hospitals and a large-scale long-term care institution and experts from the southern, eastern, northern and western regions of China, which showed good geographical representativeness), expecting to be used in wide areas. However, it was only tested for reliability and validity in a sample from a province in China limited by the small number of gerontological nurse specialist organizations and the limited resources available at that time [3]. Therefore, we first validated and revised this instrument on a national scale based on the data we collected in this study by dividing the sample into two subsamples randomly and evenly, subsample one used for confirmatory factor analysis and subsample two used for exploratory factor analysis. And then, combined with the results of confirmatory and exploratory factor analysis and expert consultation, revisions were made to the original instrument. The revised instrument consists of three domains (attitude, skill, and knowledge), nine second-level dimensions (professional self-identity, learning enthusiasm, clinical nursing skills, communication and management skills, legal/ethical practice skills, professional development skills, research and analysis decision-making skills, professional knowledge, and basic knowledge), and 49 items. It showed sound internal consistency reliability (Cronbach’s α = 0.98), test–retest reliability (Spearman coefficient = 0.87), and construct validity (the total variances explained by the factors extracted from the three domains were all greater than 80%; the model fit tested by confirmatory factor analysis was χ^2^/df < 3; SRMR ≤ 0.05; RMSEA < 0.05; and GFI, AGFI, IFI, TLI, and CFI > 0.9).

Each item in this scale was rated on a 5-point Likert scale (1 = highly inconsistent, 2 = moderately inconsistent, 3 = consistent, 4 = moderately consistent, and 5 = highly consistent). The higher the score, the stronger the core competency level. As this scale contains three subscales and the total number of items in these three subscales is different, we standardized the scores by calculating the mean value of the total scores, that is, the total score of each subscale or dimension divided by the number of items in each subscale or dimension, to easily compare the subscales or dimensions.

### Data collection procedure

The online questionnaire link was sent to nearly all gerontological nurse specialists trained by province-level or above training organizations between March 2019 and January 2020. Our previous survey showed a total of 13 province-level or above gerontological nurse specialist training organizations in China as of April 2019. Thus, we invited one to two staff members from each organization to assist in distributing the online questionnaire link to their corresponding organization’s WeChat or QQ groups, which comprise all trained gerontological nurse specialists. Two functions of the online questionnaire platform were used to ensure the completeness of the questionnaire and the response rate. The first function was to set all of the items in the online questionnaire as mandatory questions. The second function allowed participants to save their answers when they did not have sufficient time to finish the questionnaire at once. Additionally, because the survey period was long, we set the function that participants could only submit the questionnaire once to avoid filling in the questionnaire repeatedly. If all items in a questionnaire were answered identically and the time for completing the questionnaire was less than five minutes, the questionnaire was regarded as an invalid questionnaire and eliminated from the analysis.

### Statistical analysis

SPSS 19.0 (SPSS Inc., Chicago, Illinois) was used for data analysis. Respondents’ characteristics were presented as frequencies, percentages, or ${\rm{x}}\,{\rm{ \pm }}\,{\rm{SD}}$. The core competency scores were presented as medians (25% quartile and 75% quartile) because the data did not follow a normal distribution. Univariate analysis and multivariate stepwise regression analyses were combined to analyze the factors influencing participants’ core competency levels. In the univariate analysis, two-sided Mann‒Whitney U tests and Kruskal‒Wallis H tests with or without least significant difference methods for pairwise comparison were used for categorical variables, and the Spearman rank test was used for continuous variables. All variables with a P < 0.05 in the univariate analysis were entered into a multivariate stepwise regression analysis. P < 0.05 was considered statistically significant.

## Results

### Participants

The online questionnaire was completed and returned by 1939 gerontological nurse specialists (response rate = 80.72%); 208 of the questionnaires were considered invalid. Ultimately, 1731 respondents were included in the analysis. The respondents came from 30 provinces (autonomous regions or province-level municipalities) of China and worked in all levels of medical institutions and clinical departments (79.3% worked in tertiary hospitals, and 50.6% worked in geriatric departments). The median age of the participants was 33 (30, 37) years old, and the majority were female (99.1%). 83.0% of participants had a bachelor’s degree, and the mean working experience length of geriatric nursing was 7.25 ± 5.86) years. More detailed characteristics of respondents and the core competency scores for respondents with different social demographic characteristics are shown in Table 2.

**Table 2:** The core competency levels of participants with different characteristics and the univariate analysis results for core competency

Variable [(n, %) or (x ± SD)]	Attitude	Skill	Knowledge	Total scale
∎ **Individual-related factors**				
**Age (years)**				
20–29 (386, 22.3%)	4.00 (3.00, 4.57)	3.48 (3.00, 4.11)	3.71 (3.00, 4.14)	3.61 (3.04, 4.21)
30–39 (1067, 61.6%)	4.14 (3.57, 4.86) ^‡^	3.86 (3.14, 4.29) ^‡^	3.93 (3.07, 4.50) ^‡^	3.86 (3.27, 4.35)
≥ 40 (278, 16.1%)	4.29 (3.57, 5.00) ^‡^	3.89 (3.28, 4.51) ^‡^	4.00 (3.29, 4.57) ^‡^	3.98 (3.38, 4.48)
P value	< 0.001	< 0.001	< 0.001	< 0.001
**Gender**				
Female (1716, 99.1%)	4.14 (3.43, 4.86)	3.82 (3.11, 4.29)	3.93 (3.00, 4.43)	3.84 (3.22, 4.35)
Male (15, 0.9%)	3.86 (3.14, 4.43)	3.96 (3.00, 3.96)	4.14 (3.00, 4.93)	3.94 (3.10, 4.88)
P value	0.196	0.300	0.424	0.503
**Marital status**				
Single (235, 13.6%)	4.00 (3.14, 4.57)	3.54 (3.00, 4.18)	3.86 (3.00, 4.29)	3.63 (3.06, 4.3)
Married (1465, 84.6%)	4.14 (3.43, 4.86) ^‡^	3.82 (3.10, 4.29) ^‡^	3.93 (3.00, 4.43)	3.84 (3.27, 4.35)
Divorced/other (31, 1.8%)	4.29 (3.43, 5.00) ^‡^	4.1 (3.75, 4.50) ^‡§^	4.07 (3.71, 4.79) ^‡§^	4.10 (3.70, 4.57)
P value	0.009	0.008	0.032	0.004
**Education level**				
College graduated or below (260, 15.0%)	4.00 (3.14,4.71)	3.50 (3.00, 4.14)	3.68 (3.00, 4.14)	3.63 (3.05, 4.22)
Bachelor (1437, 83.0%)	4.14 (3.43,4.86) ^‡^	3.86 (3.11, 4.29) ^‡^	3.93 (3.07, 4.50) ^‡^	3.86 (3.27, 4.37)
Master or above (34, 2.0%)	4.50 (3.86,4.89) ^‡^	4.00 (3.33, 4.55) ^‡^	4.00 (3.54, 4.71) ^‡^	3.91 (3.58, 4.68)
P value	0.002	0.007	< 0.001	< 0.001
**Job title**				
None (718, 41.5%)	4.00 (3.29, 4.71)	3.79 (3.00, 4.25)	3.93 (3.00, 4.38)	3.80 (3.10, 4.35)
Nursing team leader/clinical nursing teacher (717, 41.4%)	4.14 (3.57, 4.86) ^‡^	3.79 (3.11, 4.29)	3.93 (3.21, 4.43)	3.82 (3.28, 4.36)
Vice head nurse/head nurse (239, 13.8%)	4.29 (3.57, 5.00) ^‡^	3.93 (3.18, 4.32)	4.00 (3.14, 4.50) ^‡^	3.98 (3.33, 4.33)
Department head nurse or above (57, 3.3%)	4.57 (3.86, 4.93) ^‡§^	3.86 (3.20, 4.64)	3.86 (3.29, 4.50)	3.94 (3.37, 4.56)
P value	0.001	0.150	0.168	0.074
**Professional title**				
Nurse (142, 8.2%)	3.86 (3.00, 4.43)	3.46 (3.00, 4.29)	3.54 (3.00, 4.21)	3.58 (2.98, 4.37)
Senior nurse (694, 40.1%)	4.00 (3.43, 4.71) ^‡^	3.68 (3.04, 4.14)	3.79 (3.00, 4.21)	3.71 (3.12, 4.23)
Nurse-in-charge (764, 44.1%)	4.29 (3.57, 4.86) ^‡§^	3.89 (3.18, 4.32) ^‡§^	4.00 (3.21, 4.50) ^‡§^	3.92 (3.35, 4.41)
Associate professor of nursing or above (131, 7.6%)	4.43 (3.43, 5.00) ^‡§^	3.96 (3.32, 4.46) ^‡§^	4.00 (3.36, 4.64) ^‡§^	4.00 (3.39, 4.47)
P value	< 0.001	< 0.001	< 0.001	< 0.001
**Working experience lengths of geriatric nursing** (7.25 ± 5.86 years)				
Spearman’s correlation coefficients	0.18	0.19	0.21	0.22
P value	< 0.001	< 0.001	< 0.001	< 0.001
**Attitudes toward caring for older adults** (3.58 ± 1.75 scores)				
Spearman’s correlation coefficients	0.42	0.36	0.36	0.40
P value	< 0.001	< 0.001	< 0.001	< 0.001
∎ **Employer-related factors**				
**Hospital level**				
Tertiary hospital (1372, 79.3%)	4.14 (3.46, 4.86)	3.82 (3.12, 4.29)	3.93 (3.07, 4.50)	3.86 (3.27, 4.35)
Secondary hospital (321, 18.5%)	4.00 (3.14, 4.71) ^‡^	3.68 (3.00, 4.16) ^‡^	3.71 (3.00, 4.29) ^‡^	3.71 (3.04, 4.27)
Primary health care institutions (38, 2.2%)	4.29 (3.43, 4.89)	3.93 (3.00, 4.57)	3.96 (3.00, 4.18)	4.06 (3.08, 4.40)
P value	0.006	0.133	0.027	0.023
**Department**				
Geriatrics (875, 50.6%)	4.29 (3.57, 4.86)	3.89 (3.18, 4.43)	4.00 (3.21, 4.57)	3.94 (3.29, 4.47)
Respiratory/Neurology/Cardiology (267,15.4%)	4.00 (3.43, 4.86)	3.86 (3.07, 4.18)	3.86 (3.00, 4.36) ^‡^	3.86 (3.22, 4.29)
Administration (51, 2.9%)	4.00 (3.43, 5.00)	3.71 (3.14, 4.04)	3.79 (3.00, 4.21) ^‡^	3.80 (3.16, 4.16)
Other (538, 31.1%)	4.00 (3.29, 4.57) ^‡^	3.68 (3.00, 4.07) ^‡§^	3.71 (3.00, 4.14) ^‡§^	3.71 (3.08, 4.14)
P value	0.001	< 0.000	< 0.001	< 0.001
**Whether the department had exact job responsibilities for gerontological nurse specialists**				
Yes (1121, 64.8%)	4.14 (3.43, 4.86)	3.93 (3.18, 4.43)	4.00 (3.14, 4.57)	3.94 (3.32, 4.46)
No (610, 35.2%)	4.00 (3.43, 4.71)	3.57 (3.00, 4.00)	3.71 (3.00, 4.07)	3.68 (3.08, 4.10)
P value	0.002	< 0.001	< 0.001	< 0.001
**Whether the gerontological nurse specialist certificate was associated with a priority for promotion**				
Yes (584, 33.7%)	4.29 (3.43, 5.00)	4.00 (3.32, 4.54)	4.00 (3.21, 4.64)	4.02 (3.37, 4.51)
No (1147, 66.3%)	4.00 (3.43, 4.71)	3.68 (3.04, 4.11)	3.86 (3.00, 4.29)	3.73 (3.14, 4.22)
P value	0.008	< 0.001	< 0.001	< 0.001
**Whether the certificate of gerontological nurse specialist was associated with increased salary**				
Yes (466, 26.9%)	4.21 (3.39, 5.00)	4.00 (3.32, 4.57)	4.00 (3.21, 4.57)	4.02 (3.37, 4.55)
No (1265, 73.1%)	4.00 (3.43, 4.71)	3.71 (3.04, 4.14)	3.86 (3.00, 4.36)	3.78 (3.16, 4.27)
P value	0.085	< 0.001	< 0.001	< 0.001
**The degree of satisfaction toward the attention and support given by the hospital or department** (3.77 ± 0.91 scores)				
Spearman’s correlation coefficients	0.29	0.32	0.30	0.33
P value	< 0.001	< 0.001	< 0.001	< 0.001
**The degree of satisfaction toward the learning opportunities provided by the hospital or department** (3.87 ± 0.90 scores)				
Spearman’s correlation coefficients	0.29	0.30	0.29	0.32
P value	< 0.001	< 0.001	< 0.001	< 0.001
**The degree of satisfaction toward the promotion rules set by the hospital or department** (3.39 ± 0.97 scores)				
Spearman’s correlation coefficients	0.22	0.27	0.23	0.27
P value	< 0.001	< 0.001	< 0.001	< 0.001
**The degree of satisfaction toward the position salary or allowance provided by the hospital or department** (3.21 ± 1.01 scores)				
Spearman’s correlation coefficients	0.21	0.24	0.21	0.24
P value	< 0.001	< 0.001	< 0.001	< 0.001
∎ **Training-related factors**				
**Location where gerontological nurse specialist training was received †**				
Eastern China (548, 31.7%)	4.29 (3.71, 5.00)	3.93 (3.29, 4.39)	4.00 (3.29, 4.57)	3.98 (3.43, 4.47)
Central China (343, 19.8%)	4.00 (3.14, 4.71) ^‡^	3.79 (3.00, 4.25) ^‡^	3.86 (3.00, 4.29) ^‡^	3.76 (3.08, 4.31)
Western China (840, 48.5%)	4.00 (3.43, 4.71) ^‡^	3.71 (3.07, 4.14) ^‡^	3.86 (3.00, 4.36) ^‡^	3.78 (3.22, 4.29)
P value	< 0.001	< 0.001	< 0.001	< 0.001
**The degree to which the training content met clinical needs** (4.04 ± 0.72 scores)				
Pearson’s correlation coefficients	0.22	0.27	0.29	0.30
P value	< 0.001	< 0.001	< 0.001	< 0.001
**Received continuing education related to geriatric nursing**				
Yes (937, 54.1%)	4.29 (3.57, 4.86)	3.89 (3.21, 4.39)	4.00 (3.29, 4.57)	3.96 (3.35, 4.45)
No (794, 45.9%)	4.00 (3.29, 4.71)	3.68 (3.00, 4.14)	3.79 (3.00, 4.21)	3.71 (3.10, 4.22)
P value	< 0.001	< 0.001	< 0.001	< 0.001

†According to the economic level, the Chinese government divided the country into three economic regions: the eastern, central, and western regions. Thus, the participants were assigned to these three regions according to the location where they received gerontological nurse specialist training

^‡^ indicates that in the comparison of multiple groups (Kruskal‒Wallis H tests), the scores of other groups were significantly different from the reference group (the first group). ^§^ indicates that other groups were significantly different from the second group

### Core competency level

Table 3 summarizes the core competency scores of gerontological nurse specialists. The overall core competency level of gerontological nurse specialists was 3.84. Among the subscales, the attitude subscale received the highest score (4.14), and the skill subscale received the lowest score (3.82). Among the nine dimensions, legal/ethical practice skills (4.33) and professional self-identity (4.25) received the highest score, while research and analysis decision-making skills (3.60) and professional development skills (3.25) received the lowest score.

**Table 3:** The scores of gerontological nurse specialists’ core competency

Scale	P_50_	(P_25_, P_75_)
**Total scale score**	3.84	(3.22, 4.35)
**Subscale scores**		
Attitude subscale	4.14	(3.43, 4.86)
Knowledge subscale	3.93	(3.00, 4.43)
Skill subscale	3.82	(3.11, 4.29)
**Dimension scores**		
Legal/ethical practice skills	4.33	(3.67, 5.00)
Professional self-identity	4.25	(3.75, 5.00)
Learning enthusiasm	4.00	(3.00, 5.00)
Clinical nursing skills	4.00	(3.10, 4.30)
Professional knowledge	4.00	(3.00, 4.00)
Communication and management skills	3.83	(3.00,4.33)
Basic knowledge	3.75	(3.00, 4.00)
Research and analysis decision-making skills	3.60	(3.00, 4.20)
Professional development skills	3.25	(2.75, 4.00)

### Factors associated with core competency

#### Univariate analysis results

In the univariate analysis (Table 1), all of the included variables were associated with the score of the total scale (P < 0.05) and were subsequently entered into the multivariate analysis, except for the gender and job title (P > 0.05).

#### Multivariate analysis results

The results of the multivariate stepwise regression analysis are shown in Table 4. For the included individual-related factors, only the working experience length of geriatric nursing (β = 0.092, P < 0.05) and attitudes toward caring for older adults (β = 0.239, P < 0.05) were positively associated with the core competency level of respondents.

**Table 4:** Multivariate stepwise regression analysis of gerontological nurse specialists’ core competency scores (only the variables with P < 0.05 are presented)

Dependent variables	Independent variables	B	S.E.	β	*t*	P	95% CI LB	95% CI UB	
**Total scale**	Constant	2.324	0.174		13.374	< 0.001	1.983	2.665	F = 22.33, P < 0.001; R^2^ = 0.25 Adjusted R^2^ = 0.24
	**Individual-related factors**								
	Working experience lengths of geriatric nursing	0.011	0.003	0.092	3.497	< 0.001	0.005	0.018	
	Attitudes toward caring for older adults	0.099	0.009	0.239	10.697	< 0.001	0.081	0.117	
	**Employer-related factors**								
	Departments (Geriatrics = reference)								
	Respiratory/Neurology/Cardiology department	-0.061	0.046	-0.031	-1.336	0.182	-0.151	0.029	
	Administration department	-0.248	0.096	-0.058	-2.582	0.010	-0.437	-0.060	
	Other departments	-0.115	0.038	-0.074	-3.030	0.002	-0.189	-0.040	
	Whether the department had exact job responsibilities for gerontological nurse specialists (Yes = reference)								
	No	-0.083	0.034	-0.055	-2.436	0.015	-0.150	-0.016	
	The degree of satisfaction toward the attention and support given by hospital or department leaders	0.090	0.024	0.114	3.709	< 0.001	0.042	0.138	
	The degree of satisfaction toward the promotion rules set by hospital or department leaders	0.091	0.022	0.122	4.049	< 0.001	0.047	0.135	
	**Training-related factors**								
	Economic zone where training organizations are located (Eastern = reference)								
	Central	-0.147	0.046	-0.081	-3.194	0.001	-0.237	-0.057	
	Western	-0.087	0.036	-0.060	-2.397	0.017	-0.157	-0.016	
	The degree to which the training content met clinical needs	0.137	0.023	0.136	6.080	< 0.001	0.093	0.182	
**Attitude subscale**	Constant	2.633	0.208		12.632	< 0.001	2.224	3.042	F = 15.08, P < 0.001; R^2^ = 0.18 Adjusted R^2^ = 0.16
	**Individual-related factors**								
	Working experience lengths of geriatric nursing	0.012	0.004	0.074	2.707	0.007	0.003	0.02	
	Attitudes toward caring for older adults	0.144	0.012	0.275	11.703	< 0.001	0.119	0.168	
	**Employer-related factors**								
	The degree of satisfaction toward the attention and support given by hospital or department leaders	0.090	0.037	0.090	2.405	0.016	0.017	0.163	
	**Training-related factors**								
	Economic zone where training organizations are located (Eastern = reference)								
	Central	-0.203	0.060	-0.089	-3.361	0.001	-0.322	-0.085	
	Western	-0.123	0.048	-0.068	-2.592	0.01	-0.217	-0.03	
	The degree to which the training content met clinical needs	0.073	0.030	0.057	2.421	0.016	0.014	0.132	
**Skill subscale**	Constant	2.479	0.185		13.373	< 0.001	2.115	2.842	F = 20.53, P < 0.001; R^2^ = 0.21, Adjusted R^2^ = 0.20
	**Individual-related factors**								
	The attitudes toward caring for older adults	0.094	0.010	0.211	9.256	< 0.001	0.074	0.114	
	**Employer-related factors**								
	Departments (Geriatrics = reference)								
	Respiratory/Neurology/Cardiology department	-0.072	0.049	-0.033	-1.451	0.147	-0.168	0.025	
	Administration department	-0.223	0.103	-0.049	-2.165	0.031	-0.426	-0.021	
	Other departments	-0.138	0.039	-0.082	-3.521	< 0.001	-0.215	-0.061	
	Whether the department had exact job responsibilities for gerontological nurse specialists (Yes = reference)								
	No	-0.086	0.040	-0.053	-2.176	0.030	-0.164	-0.009	
	The degree of satisfaction toward the attention and support given by hospital or department leaders	0.091	0.027	0.107	3.413	0.001	0.039	0.144	
	The degree of satisfaction toward the promotion rules set by hospital or department leaders	0.089	0.026	0.111	3.419	0.001	0.038	0.14	
	**Training-related factors**								
	Economic zone where training organizations are located (Eastern = reference)								
	Central	-0.155	0.051	-0.080	-3.054	0.002	-0.255	-0.056	
	Western	-0.103	0.040	-0.066	-2.589	0.01	-0.18	-0.025	
	The degree to which the training content met clinical needs	0.140	0.025	0.129	5.635	< 0.001	0.091	0.188	
**Knowledge subscale**	(Constant)	2.319	0.191		12.117	< 0.001	1.943	2.694	F = 22.65, P < 0.001; R^2^ = 0.22 Adjusted R^2^ = 0.21
	**Individual-related factors**								
	Attitudes toward caring for older adults	0.093	0.010	0.206	9.103	< 0.001	0.073	0.113	
	**Employer-related factors**								
	Departments (Geriatrics = reference)								
	Respiratory/Neurology/Cardiology department	-0.140	0.050	-0.064	-2.815	0.005	-0.237	-0.042	
	Administration department	-0.316	0.104	-0.068	-3.056	0.002	-0.519	-0.113	
	Other departments	-0.228	0.040	-0.135	-5.702	< 0.001	-0.306	-0.149	
	Whether the department had an exact position statement for gerontological nurse specialists (Yes = reference)								
	No	-0.115	0.038	-0.07	-3.073	0.002	-0.189	-0.042	
	The degree of satisfaction toward the attention and support given by hospital or department leaders	0.085	0.027	0.100	3.193	0.001	0.033	0.138	
	The degree of satisfaction toward the promotion rules set by hospital or department leaders	0.074	0.025	0.092	3.005	0.003	0.026	0.123	
	**Training-related factors**								
	Economic zone where training organizations are located (Eastern = reference)								
	Central	-0.181	0.050	-0.092	-3.588	< 0.001	-0.28	-0.082	
	The degree to which the training content met clinical needs	0.174	0.025	0.159	6.986	< 0.001	0.125	0.222	

CI, confidence interval; LB: lower bound; UB, upper bound

For the included employer-related factors, respondents who worked in administration (β = -0.058, P = 0.01) and other departments (β = -0.074, P = 0.002) had lower core competency scores than those who worked in the geriatrics department. Respondents who worked in departments that did not have exact job responsibilities for gerontological nurse specialists (β = -0.055, P = 0.015) were also associated with a lower core competency score. In addition, participants who were more satisfied with the attention and support given by the hospital or department (β = 0.114, P < 0.001) and who were more satisfied with the promotion rules set by the hospital or department (β = 0.122, P < 0.001) were associated with a higher core competency score.

For the included training-related factors, the economic zone where training organizations are located (central China: β = -0.081, P = 0.001; western China: β = -0.06, P = 0.017) and the degree to which the training content met clinical needs (β = 0.136, P < 0.001) were independently associated with the core competency level.

The common factors associated with the scores of three subscales (attitude, skill, and knowledge) included attitudes toward caring for older adults, the degree of satisfaction toward the attention and support given by the hospital or department, the economic zone where training organizations are located, and the degree to which the training content met clinical needs.

## Discussion

### Status of the core competency of gerontological nurse specialists

This study is the first large-scale national study to investigate gerontological nurse specialists’ core competency level in China. The results of the self-administered questionnaire showed that the core competency level of gerontological nurse specialists in China is at an above-average level, which is slightly greater than our previous survey conducted in a province in western China [20]. The attitude subscale received the highest score among the three subscales, and the scores of the two dimensions it contains (i.e., professional self-identity and learning enthusiasm) ranked in the top three in all dimensions, indicating that gerontological nurse specialists engaged passionately in geriatric nursing work. This may be the result of the efforts of the Chinese government and geriatric nursing educators in recent years [28–30], and it is also a driving force for the future development of gerontological nurse specialists.

However, the scores for professional development skills ranked last in all dimensions. Professional development is closely associated with nurses’ job satisfaction and employment retention [31]. Although gerontological nurse specialists were enthusiastic about this new nursing role during the survey period, if they encounter difficulties with obtaining promotions or they do not have sufficient abilities to develop this new nursing role, it may be difficult to maintain the team of gerontological nurse specialists. Therefore, in the early stage of developing a nursing role, it is also necessary for nursing educators and administrators to pay attention to exploring the path to professional development and improving nurses’ professional development skills to ensure the sustainable and powerful development of the new nursing role. Mijares et al. suggested that mentoring is an approach to addressing professional development. Their study demonstrated that a mentorship program with an established clinical ladder could increase work engagement and satisfaction [31], which may provide a prospect for enhancing the professional development skills of gerontological nurse specialists.

In addition, the score for research and analysis decision-making skills was comparatively low, which is similar to studies conducted on registered nurses in Saudi Arabia and Korea [32, 33] or nurse specialists in other nursing fields in China [34, 35], demonstrating that low research and analysis decision-making skills in the subcategories of nursing core competency is a common problem. As gerontological nurse specialists are the mainstay in geriatric nursing, it is essential for them to have sufficient research skills and to be able to transfer the research results to clinical practice to help clinical decision-making. However, these skills are often systematically trained in nursing postgraduate education. In this study, over 80% of gerontological nurse specialists held a bachelor’s degree or below, while a survey conducted on advanced practice registered nurses in the United States showed that 84.7% held a master’s degree [36]. It may take many years to popularize nursing education to postgraduate education, especially for developing countries. Therefore, at this stage, targeted continuing education or encouraging gerontological nurse specialists to upgrade their education level may be feasible and effective way to improve research and analysis decision-making skills.

### Factors influencing the core competency of gerontological nurse specialists

The multivariate analysis results in this study showed that individual-, employer-, and training-level factors were associated with gerontological nurse specialists’ core competency level. The individual-level factors included the working experience length of geriatric nursing and attitudes toward caring for older adults. The longer gerontological nurse specialists work in geriatric nursing, the stronger their core competency, which is similar to the results of nurse specialists in other nursing fields [37, 38]. The more passionately gerontological nurse specialists care for older adults, the stronger their core competency. This may result from gerontological nurse specialists, who are passionate about caring for older adults, being more willing to learn geriatric knowledge and techniques to constantly improve their abilities to meet the health needs of older adults. Additionally, although most participants in this study held a positive attitude toward caring for older adults, approximately 10% of participants held a negative or neutral attitude. A negative or neutral attitude may affect the quality of nursing for older adults and the determination to continue to engage in geriatric nursing. Therefore, when selecting nurses to become gerontological nurse specialists, geriatric nursing managers and educators should consider incorporating the indicator of nurses’ attitudes toward caring for older adults into the selection criteria.

Employer-level factors included the department, degree of satisfaction toward the attention and support and promotion rules given or set by the hospital or department, and whether the department had exact job responsibilities for gerontological nurse specialists. Our study showed that the core competency levels of gerontological nurse specialists working in geriatrics, respiratory or neurology, or cardiology departments were higher than those working in administration or other departments. The former departments usually have comparatively more older patients than the latter departments, which correspondingly could provide more opportunities for gerontological nurse specialists to practice their skills and knowledge. Therefore, it is necessary to provide more opportunities for gerontological nurse specialists working in departments with fewer older adult patients to maintain their core competency. In addition, it is noteworthy that 2.9% of gerontological nurse specialists work in administration departments instead of clinical departments. This may result in the waste of gerontological nurse specialists because they are scarce, and their main task is to provide clinical nursing for older adults [39].

In addition to the department, there is a paucity of studies exploring other employer-level factors associated with gerontological nurse specialists’ or other geriatric nurses’ core competencies. Cheng’s study showed that performance bonuses were associated with the level of competency of nursing assistants in long-term care institutions [40]. Negrin’s integrative review found that funding, time and professional development resources, and leaders’ attitudes affect undergraduate nurse educators’ knowledge, skills, or attitudes about older adults and their care [41]. Similar results were obtained in this study. Therefore, it is urgent to explore a suitable post-management system for gerontological nurse specialists.

The training-associated factors were the economic zone where training organizations are located and the degree to which the training content met clinical needs. The economic level is a generally accepted factor that affects education level, while the economic level of the region where the training organizations are located is difficult to change. Therefore, we suggest that nursing educators and administrators try to unify the training programs and admission requirements for both gerontological nurse specialists and coachers among training organizations to minimize the differences caused by economic level as much as possible. Regarding the degree to which the training content met clinical needs, our previous study showed that gerontological nurse specialists expected to extend the training time to more than three months and to strengthen the training content in the aspects of professional knowledge and techniques of geriatric nursing as well as research skills [42]. Therefore, extending the training length or providing targeted continuing education courses based on nurses’ needs might be helpful for them to obtain sufficient updated knowledge and skills to meet clinical needs.

### Limitations

This study also has several limitations. First, we only surveyed gerontological nurse specialists trained by province-level or above training organizations, and those trained by other training organizations were not included in this study. Therefore, the generalization of the results of this study is limited, and the results should be carefully interpreted when applied to gerontological nurse specialists trained by other training organizations. Second, because this is only a preliminary study to explore the factors affecting the core competence of gerontological nurse specialists, we used a single-item Likert-type scale to measure some variables, e.g., attitudes toward caring for older adults and the degree to which the training content met clinical needs, which should be better measured by scales with multiple items; thus, this may lead to unstable measurement results that affect the analysis result. However, the results of this study might provide directions for further in-depth studies on the factors influencing nurses’ core competence with more reliable and valid scales. Finally, most studies showed that education level could affect nurses’ competency. However, in this study, the multivariate stepwise regression analysis results showed that the education level did not significantly influence the core competency of gerontological nurse specialists, which may result from the highly uneven distribution of sample size among groups at the education level.

## Conclusion

This national cross-sectional study showed that gerontological nurse specialists’ core competency level in China is at an above-average level. Their professional development skills and research and analysis decision-making skills are comparatively low and need further improvement. Additionally, individual-, employer-, and training-level factors could influence gerontological nurse specialists’ core competency level. The results of this study could provide a reference for geriatric nursing educators and administrators to implement targeted strategies to improve gerontological nurse specialists’ core competency level and to better manage and utilize them.

## Data Availability

The data used to support the findings of this study are available from the corresponding author upon reasonable request.

## List of abbreviations

AGFI: Adjusted goodness of fit index
CFI: Comparative fit index
CI: Confidence interval
GFI: Goodness of fit index
IFI: Incremental fit index
LB: Lower bound
RMSEA: Root mean square error of approximation
SRMR: Standardized root mean square residual
TLI: Tucker–Lewis index
UB: Upper bound
